# Axonal-Variant Guillian-Barre Syndrome Temporally Associated With mRNA-Based Moderna SARS-CoV-2 Vaccine

**DOI:** 10.7759/cureus.18291

**Published:** 2021-09-26

**Authors:** Vikas Dalwadi, Diana Hancock, Ahmad A Ballout, Anthony Geraci

**Affiliations:** 1 Neurology, Donald and Barbara Zucker School of Medicine at Hofstra/Northwell, Manhasset, USA

**Keywords:** sars-cov-2, covid-19, gbs following covid-19 vaccine, mrna-1273 vaccine, gbs variant

## Abstract

We present a case of an 86-year-old woman who presented with a progressive quadriparesis two days after her second dose of Moderna SARS-CoV-2 vaccine, with cerebrospinal fluid (CSF) evidence of cytoalbuminocytological dissociation and electromyogram/nerve conduction studies (EMG/NCS) findings suggestive of acute axonal motor neuropathy. Her clinical symptoms did not improve with plasmapheresis.

There appears to be a potential temporal association between the inoculation of mRNA-based SARS-CoV-2 vaccines and the development of Guillain-Barre Syndrome (GBS). Despite this possible association, infection prevention using highly effective mRNA-based vaccines remains highly recommended. Large epidemiological studies of SARS-CoV-2 vaccine-related adverse events are needed. Physicians should be aware of this possible temporal association since the prompt diagnosis and treatment of GBS can drastically improve outcomes.

The aim is to report a case of axonal-variant GBS that was temporally associated with an mRNA-based SARS-CoV-2 vaccine.

## Introduction

Guillain-Barre syndrome (GBS) is an immune-mediated polyradiculoneuropathy that often presents as an acute paralyzing illness preceded by an infection, or less commonly, immunizations [1]. Various case reports of GBS after SARS-CoV-2 infections raised the concern of a possible post-infectious association; however, a recently published large epidemiological study failed to demonstrate a statistically significant correlation [2]. Aside from the 1976 swine influenza vaccine, which demonstrated an increased risk of post-vaccination GBS [3], no other vaccines have been shown to have a statistically significant association with the development of GBS, including the SARS-CoV-2 vaccine. There were very few cases of GBS observed in the safety trials for all SARS-CoV-2 vaccines and only two reported cases in clinical practice. We present a case of axonal-variant GBS that was temporally associated with the administration of an mRNA-based SARS-CoV-2 vaccine.

## Case presentation

An 86-year-old woman with atrial fibrillation, diabetes mellitus type 2, aortic stenosis, and ischemic stroke with no residual deficits presented with lower extremity weakness and pain. Her neurological examination progressed from a subtle paraparesis to a dense lower-extremity predominant quadriparesis within one week. She had received the second dose of the Moderna SARS-CoV-2 vaccine two days prior to her onset of symptoms. Cerebrospinal fluid (CSF) analysis demonstrated albuminocytological dissociation (WBC < 1 cells/µL and protein of 129 mg/dL) consistent with GBS. The remainder of her CSF immunological studies was negative including protein electrophoresis, gram stain, glucose, viral PCR panel, and paraneoplastic antibody evaluation. Immunoserologies were negative including thyroid function tests and anti-ganglioside antibodies. Confirmatory electromyography and nerve conduction studies (NCS) were performed which demonstrated low amplitude right peroneal, tibial, and ulnar motor evoked responses with normal distal latencies, segmental conduction velocities, and sensory responses, consistent with acute axonal motor neuropathy. She was treated with plasmapheresis for five sessions without notable improvement in her strength.

## Discussion

This case highlights a patient who developed a progressive quadriparesis two days after receiving the second dose of the Moderna mRNA-based SARS-CoV-2 vaccine, in line with the time course of previously reported cases of post-vaccine associated GBS [3]. The rapid progression of weakness, albuminocytological dissociation, and low amplitude motor evoked responses on NCS are all consistent with axonal-variant GBS.

Overall, there were very few neuroinflammatory events reported across all of the major SARS-CoV-2 vaccine trials, which used various platforms. Two cases of GBS occurred ten days after injection with both placebo and vaccine in the clinical trial for Johnson & Johnson’s vector-based vaccine [4]. There were three cases of Bell’s Palsy reported in the clinical trial for the mRNA-based Moderna vaccine [5], and three cases of transverse myelitis in the clinical trial for the vector-based AstraZeneca vaccine [6]. There were no reported neuroinflammatory events in the Pfizer-BioNTech phase 2/3 trial [7].

There have been only a few reports of GBS after the administration of SARS-CoV-2 vaccines in clinical practice. One report involved a patient that developed symptoms two weeks after the administration of the Pfizer-BioNtech vaccine [8], and a second case involved a patient with a previous history of GBS years earlier that presented with symptoms eight days after receiving a vector-based vaccine [9]. Albuminocytological dissociation was evident in both cases. The diagnosis was confirmed with evidence of lumbosacral nerve root enhancement in the first case, and nerve conduction block on the NCS in the second case. The NCS findings in the second case were consistent with a demyelinating pattern, a far more common pattern in GBS than the axonal pattern seen in our patient [10].

## Conclusions

While causality between vaccine administration and the development of GBS in our patient cannot be proved, the onset of symptoms is consistent with previously reported cases of post-vaccination GBS. Overall, there have been very few cases of GBS reported after the administration of SARS-CoV-2 vaccines in all the safety trials, and only two reported cases in clinical practice to date. In the absence of large epidemiological studies of SARS-CoV-2 vaccine-related adverse events, the incidence of GBS after vaccine administration remains uncertain, although the paucity of cases suggest that it is likely rare. Since infections in general, including SARS-CoV-2, may trigger GBS, infection prevention using the highly effective mRNA-based SARS-CoV-2 vaccines remain highly recommended. Physicians should be aware of the possible temporal association between the SARS-CoV-2 vaccine and GBS since the prompt diagnosis and treatment of GBS can drastically improve outcomes.

## References

[1] Pritchard J (2010). Guillain-Barré syndrome. Clin Med (Lond).

[2] Keddie S, Pakpoor J, Mousele C (2021). Epidemiological and cohort study finds no association between COVID-19 and Guillain-Barré syndrome. Brain.

[3] Souayah N, Yacoub HA, Khan HM (2012). Guillain-Barré syndrome after influenza vaccination in the United States, a report from the CDC/FDA vaccine adverse event reporting system (1990-2009). J Clin Neuromuscul Dis.

[4] Sadoff J, Le Gars M, Shukarev G (2021). Interim results of a phase 1-2a trial of Ad26.COV2.S Covid-19 vaccine. N Engl J Med.

[5] Baden LR, El Sahly HM, Essink B (2021). Efficacy and safety of the mRNA-1273 SARS-CoV-2 vaccine. N Engl J Med.

[6] Voysey M, Clemens SA, Madhi SA (2021). Safety and efficacy of the ChAdOx1 nCoV-19 vaccine (AZD1222) against SARS-CoV-2: an interim analysis of four randomised controlled trials in Brazil, South Africa, and the UK. Lancet.

[7] Polack FP, Thomas SJ, Kitchin N (2020). Safety and efficacy of the BNT162b2 mRNA Covid-19 vaccine. N Engl J Med.

[8] Waheed S, Bayas A, Hindi F, Rizvi Z, Espinosa PS (2021). Neurological cmplications of COVID-19: Guillain-Barre syndrome following Pfizer COVID-19 vaccine. Cureus.

[9] Finsterer J (2021). Exacerbating Guillain-Barré syndrome eight days after vector-based COVID-19 vaccination. Case Rep Infect Dis.

[10] Shang P, Zhu M, Wang Y (2021). Axonal variants of Guillain-Barré syndrome: an update. J Neurol.

